# Different stability of social-communication problems and negative demanding behaviour from infancy to toddlerhood in a large Dutch population sample

**DOI:** 10.1186/1753-2000-8-19

**Published:** 2014-07-07

**Authors:** Esmé Möricke, GA Martijn Lappenschaar, Sophie HN Swinkels, Nanda NJ Rommelse, Jan K Buitelaar

**Affiliations:** 1 Department of Psychiatry, Nijmegen Centre for Evidence-Based Practice, Radboud University Nijmegen Medical Centre, P.O. Box 9101, 6500 HB Nijmegen, The Netherlands; 2 Department of Cognitive Neuroscience, Donders Institute for Brain, Cognition and Behaviour, Radboud University Nijmegen Medical Centre, P.O. Box 9104, 6500 HE Nijmegen, The Netherlands; 3 Karakter Child and Adolescent Psychiatry University Centre, Reinier Postlaan 12, 6525 GC Nijmegen, The Netherlands

**Keywords:** Factor mixture modelling, Behavioural and developmental profiles and problems, Continuity and stability, Infants and toddlers, General population

## Abstract

**Background:**

Little is known about the stability of behavioural and developmental problems as children develop from infants to toddlers in the general population. Therefore, we investigated behavioural profiles at two time points and determined whether behaviours are stable during early development.

**Methods:**

Parents of 4,237 children completed questionnaires with 62 items about externalizing, internalizing, and social-communicative behaviour when the children were 14–15 and 36–37 months old. Factor mixture modelling identified five homogeneous profiles at both time points: three with relatively normal behaviour or with mild/moderate problems, one with clear communication and interaction problems, and another with pronounced negative and demanding behaviour.

**Results:**

More than 85% of infants with normal behaviour or mild problems at 14–15 months were reported to behave relatively typically as toddlers at 36–37 months. A similar percentage of infants with moderate communication problems outgrew their problems by the time they were toddlers. However, infants with severe problems had mild to severe problems as toddlers, and did not show completely normal behaviour. Improvement over time occurred more often in children with negative and demanding behaviour than in children with communication and interaction problems. The former showed less homotypic continuity than the latter.

**Conclusions:**

Negative and demanding behaviour is more often transient and a less specific predictor of problems in toddlerhood than communication and interaction problems.

## Background

Psychiatric disorders, such as those defined by the Diagnostic and Statistical Manual of mental disorders (DSM-IV-TR) [1] and the International Statistical Classification of Diseases and related health problems (ICD-10) [2], are often preceded by dysfunctioning in the first years of life [3-5], and investigators are becoming increasingly aware that, in order to understand why and how psychiatric disorders occur, it is important to look for relevant signs as early as possible, in infancy. A major barrier to this is that the DSM-IV-TR and the ICD-10 are not suitable for studying behavioural and developmental problems in children younger than 2 years, because at this age there are no specific criteria and categories for the majority of psychiatric disorders and their precursors [6,7]. In addition, these classification systems, as well as the Diagnostic Classification of mental health and developmental disorders of infancy and early childhood (DC 0-3R) [8], contain fixed algorithms that offer few possibilities for classifying children who score just below the diagnostic cut-off (milder cases), but who may be at serious risk for later disorders. For instance, severe social-communication problems, which are characteristic for autism spectrum disorder (ASD), may be apparent and lead to a reliable diagnosis before 2 years of age, whereas less pronounced problems are only recognized later [9]. This hinders the investigation of the continuity of psychiatric dysfunctioning over time.

A statistical approach may be an alternative way to investigate coherent patterns of behaviour and their stability from infancy to toddlerhood and may obviate the limitations of regular classification systems. Factor mixture modelling (FMM) [10] combines a common factor analysis (FA) with a latent class analysis (LCA) [11]. FA makes the determination of the unobserved factors underlying the observed variables possible. LCA, based on an empirically bottom-up approach, enables the classification of children into mutually exclusive groups on the basis of the type and/or severity of behaviour. The advantage is that not only groups with deviant behaviour, but also with only mild problems or without problems can be formed, which provides a better overall view of symptom severity. Thus, FMM gives insight in both the clustering of items into factors and the grouping of individuals into classes representing all possible dimensions. Application of this method at several time points makes it possible to distinguish groups of children with different developmental patterns [12]: stable without problems, transitory problems, late-onset problems, and stable with problems, either the same problems (homotypic continuity) or different problems (heterotypic continuity) [13,14].

The preferred way to study changes in behaviour over time is to use a longitudinal, large-scale population-based design, because this design is the least biased with regard to frequency of disorders, symptom severity, and level of impairment. In addition, specific diagnostic algorithms can be used, adjusted for age or developmental level [3,15]. However, there have been only a few prospective studies focusing on the prevalence and stability of behavioural and emotional problems in infants and toddlers.

Briggs-Gowan et al. [14] studied the stability of social-emotional and behavioural problems over 1 year in infants and toddlers and found half of their sample to have persistent psychopathology. Homotypic persistence rates were about 38% for internalizing behaviour, 50% for externalizing behaviour, and 39% for dysregulation. Heterotypic persistence was considerably lower (12%). Mathiesen and Sanson [12] found that nearly 12% of children had problems of emotional adjustment, social adjustment, overactive-inattentive behaviour, and regulation at both 18 and 30 months of age. However, the type of stability was only determined within each separate factor, and not between various factors, so the study did not provide information about heterotypic continuity. In a follow-up study of the same sample [16], the authors found that undercontrolled problems decreased and internalizing problems increased up to age 4.5 years; however, the number of items was limited and only these two types of symptoms were considered. Bufferd, Dougherty, Carlson, Rose, and Klein [17] assessed psychiatric disorders in preschoolers. Having a psychiatric diagnosis at 3 years led to a fivefold greater risk of having such a diagnosis at 6 years, and 14% of the children met criteria at both time points. Homotypic continuity occurred for anxiety, attention-deficit/hyperactivity disorder (ADHD), and oppositional defiant disorder (ODD), whereas heterotypic continuity existed between anxiety and depression, anxiety and ODD, and ADHD and ODD. Beyer, Postert, Müller, and Furniss [18] investigated the continuity of, and the changes in, two types of symptoms over a 4-year period from preschool to primary school. The continuity of internalizing symptoms (37%) was higher than that of externalizing symptoms (19%), but there was substantial crossover from externalizing to internalizing symptoms (15%) and from externalizing symptoms to a combination of both types of problems (18%). The authors also reported that 86% of children without mental health problems at preschool did not have such problems at primary school. Further evidence for the stability of preschool behavioural and emotional problems in relation to psychopathology in childhood and adolescence exists [3,19].

Previous population-based studies included up to 1,000 participants, but mainly focused on clusters of variables and used cut-off values to classify children into two groups (with or without problems), which resulted in a loss of information. Moreover, emphasis was often on deviant and problematic behaviour, and normal behaviour and improvement of functioning were not always considered. Previously, we investigated normal and deviant behaviour in a population-based sample involving 6,330 infants aged 14–15 months by combining a dimensional and categorical approach [20]. Parents answered items about externalizing, internalizing, and social-communicative behaviour which could be divided over nine factors, namely deviant communication, negative emotionality, deviant reactive behaviour, deviant play behaviour, demanding behaviour, social anxiety/inhibition, advanced social interaction problems, basic social interaction problems, and sleep problems. LCA identified five homogeneous profiles, three of which were indicative of increased problems: one was related to moderate communication problems, another to severe communication and social interaction problems, and the last to severe negative and demanding behaviour. Thus, certain behavioural and developmental profiles can be recognized at the age of 14–15 months, but the key question is how stable these profiles are. The aim of the current study was to explore the stability of normal, externalizing, internalizing, and social-communicative behaviour from infancy to toddlerhood. To this end, we investigated (1) which homogeneous profiles can be identified in these children at the age of 36–37 months, and (2) to what extent these profiles are stable passing from infancy to toddlerhood.

## Methods

### Participants

The Medical Ethics Committee of the University Medical Centre Utrecht approved the study. We used a subsample from a birth cohort of children born between August 2000 and August 2001 in the province of Utrecht, The Netherlands (*N* = 12,297). Parents received two questionnaires concerning infant behaviour and development: one at T1, when their child was 14–15 months old (*M* = 14.70; *SD* = 0.68), and another at T2, when their child was 36–37 months old (*M* = 36.64; *SD* = 2.63). Parents who returned the questionnaires automatically consented to participate. Children were included if they had maximally six missing values (<10% of 62 items) on each questionnaire (thus at both time points), resulting in 4,237 participants (i.e., a response rate of 34.5% of 12,297 children eligible). At both time points, the questionnaire was mainly completed by mothers: T1 mothers 84.4% (*n* = 3,575), fathers 10.2% (*n* = 431), both parents 0.3% (*n* = 14), and unknown respondent 5.1% (*n* = 217); T2 mothers 89.2% (*n* = 3,779), fathers 9.3% (*n* = 392), both parents 0.3% (*n* = 13), and unknown respondent 1.3% (*n* = 53). In at least 82.1% of the cases the respondent was the same at T1 and T2. The sample consisted of 2,176 boys (51.4%) and 2,061 girls (48.6%). Most children were developing normally, as evaluated by the parents. Of all children, 54 (1.3%) had a mental or physical handicap, 176 (4.2%) had a physical disease, and 286 (6.8%) used medication; health information was missing for 6 children (0.1%). They were all included in the analyses, because we wanted to explore the behaviour of all types of children.

Because access to information about non-responders was not allowed, we investigated the possibility of selection bias by comparing the data of responders with demographic data for the general population [21]. Classified according to nationality, the majority of the sample was Dutch (94.6%, *n* = 4,009), with smaller groups of other minorities: 1.4% other European (*n* = 60), 1.6% Moroccan or Turkish (*n* = 67), and 2.0% others (*n* = 84); the nationality of 0.4% of the sample was not known (*n* = 17). More than 97.5% of the children belonged to the Caucasian race, so the chance that racial differences played a meaningful role was limited. Our sample contained more Dutch children than the population average (82.1%). More parents in this sample had a high educational level (college or university degree) than in the general population (mothers: 45.4% versus 38.9%; fathers: 46.6% versus 36.0%). The socioeconomic status (SES), based on mean level of education and occupation of both parents, varied from low (*n* = 477; 11.3%) through moderate (*n* = 1,668; 39.4%) to high (*n* = 2,069; 48.8%); in 0.5% (*n* = 23) of the cases SES was unknown.

### Instruments

#### ***Utrecht Screening Questionnaire***

The Utrecht Screening Questionnaire (USQ) [22], which is completed at age 14–15 months (T1), was specially developed by a multidisciplinary panel of experts with clinical and research experience with infants and toddlers. The panel selected 79 items from a large pool of potentially interesting and relevant items from well-validated instruments, namely, the Child Behavior Checklist 1½-5 [23], the Infant-Toddler Social and Emotional Assessment [24], the Vineland Social-Emotional Early Childhood Scales [25], and the Early Screening of Autistic Traits Questionnaire (ESAT) [26,27]. The two selection criteria were that the items were specific for externalizing, internalizing, or social-communicative problems, and that they were suitable for children younger than 18 months. Subsequently, we excluded 17 less relevant items: 12 items were rather identical to other items in the same questionnaire, and 5 items were related more to aspects of parental child rearing than to child behaviour. In total 62 items were left (Table 1). Fourteen ESAT items were scored on a yes or no scale (corresponding with scores 0 or 1) and the other 48 items were rated on a three-point Likert scale (0 ‘not at all true’, 1 ‘somewhat/sometimes true’, 2 ‘clearly/often true’). See Möricke et al. [20] for a more detailed description of previous analyses.

**Table 1:** **Proportion of children with deviant scores on USQ items in exploratory factor analysis with promax rotation at T1 (*N*** **= 4,237)**

**Item USQ**^**a**^	**Factor USQ**^**a**^	**Proportion of children with deviant score**	**Cronbach’s alpha**
	**1 Deviant communication**		0.69
47^b^	Uses gestures appropriately to express him/herself	16.0	
48^b^	Points at things to show^c^	6.2	
46^b^	Understands at least ten words^c^	9.4	
45^b^	Imitates simple gestures^c^	4.6^d^	
75^b^	Uses common names like ‘mummy/daddy’^c^	6.0	
14^b^	Gives or shows something to somebody	2.8	
74^b^	Imitates sounds made by parents^c^	3.1	
76^b^	Reacts when name is called	5.8^d^	
41^b^	Pays attention when being spoken to	10.1	
	**2 Negative emotionality**		0.81
78	Is stubborn, sullen or irritable	3.0	
73	Is fussy, whiny	1.9	
66	Is extremely loud	1.2	
65	Is uncooperative	2.4	
72	Changes mood suddenly	4.1	
58	Screams a lot	4.0	
38	Is easily upset	6.7	
31	Cries a lot	2.5	
27	Cannot sit still; is restless or hyperactive	9.8	
64	Seems unhappy without clear reason	4.7^d^	
69	Bites, hits or kicks others	0.9	
54	Hurts animals or persons	1.5	
24	Cannot concentrate or pay attention for long	8.0	
57	Refuses to play active games him/herself	1.2	
30	Wants help constantly	2.5	
60	Will not share toys or other things	5.7	
	**3 Deviant reactive behaviour**		0.00
19^b^	Reacts when being spoken to	0.3	
9^b^	Reacts normally to sensory stimuli	0.2	
	**4 Deviant play behaviour**		0.20
6^b^	Plays with different toys/objects	0.3	
7^b^	Plays in various ways	1.3	
10^b^	Shows clear facial expressions	1.1	
	**5 Demanding behaviour**		0.51
34	Demands must be met immediately	13.6	
28	Cannot stand waiting; wants everything now	15.7	
22	Has angry moods	9.5	
67	Wants a lot of attention	12.8	
	**6 Social anxiety/inhibition**		0.03
15^b^	Shows interest in other children/adults	0.6	
42	Is afraid of certain animals, things or places	3.7	
18^b^	Likes to play games with others	0.7	
	**7 Advanced social interaction problems**		0.49
53^b^	Shows that he/she distinguishes parents from others	9.7^d^	
70^b^	Babbles or makes noises spontaneously^c^	5.7^d^	
61^b^	Utters sounds of joy	6.8^d^	
26^b^	Shows interest in new objects/persons	7.1^d^	
79^b^	Follows with eyes when someone moves^c^	8.8^d^	
71^b^	Stops wailing when being spoken to	5.6	
43^b^	Reaches when he/she wants to be held	1.9	
35^b^	Enjoys learning new things	2.2	
	**8 Basic social interaction problems**		0.18
17^b^	Directs social smile to parents and others	0.4	
11^b^	Makes eye contact easily	1.9	
16^b^	Likes cuddling	4.2	
13	Repeats stereotypic movements	5.5	
	**9 Sleep problems**		0.32
39	Cannot sleep alone	4.1	
63	Finds it difficult to fall asleep	7.0	
51^b^	Clings on when he/she wants to be held	3.4	

^a^Total number of items was 62. Only items with factor loading ≥ 0.30 were included (52). Other items were omitted (10): 8 Emotions are understandable; 12 Asks attention when being alone; 23 Is accident prone; 29 Keeps on trying; 32 Wants to do things him/herself; 49 Does not eat well; 52 Sits still for five minutes during reading; 56 Quickly shifts from one activity to another; 62 Cries, stays at place, waits for parent when scared; 68 Uses objects for imaginative play.

^b^Items were reversely coded.

^c^Adjusted or alternative items.

^d^Proportions of children with score ‘1’ and ‘2’ were combined and considered as deviant (<limit of 10%); in all other cases only a score of 2 represented deviant behaviour.

#### ***Social Behaviour Questionnaire***

The Social Behaviour Questionnaire (SBQ), which is completed at age 36–37 months (T2), focuses on the externalizing, internalizing, and social-communicative behaviour of toddlers. It consists of 62 items scored on a three-point Likert scale: 54 items were formulated exactly the same as in the USQ, but 6 items were adapted to the higher level of functioning expected of toddlers in comparison with infants, and 2 items were new (Table 2). The answer possibilities of the 14 ESAT items changed from yes/no to the three-point Likert scale.

**Table 2:** **Proportion of children with deviant scores and factor loadings on SBQ items in factor mixture model at T2 (*N*** **= 4,237)**

**Item SBQ**^**a**^	**Factor SBQ**^**a**^	**Proportion of children with deviant score**	**Factor loading**	**Cronbach’s alpha**
	**1 Language problems**			0.76
117^b^	Talks in full sentences^c^	2.5	0.906	
131^b^	Speaks intelligibly^c^	1.7	0.860	
143^b^	Participates in reciprocal social interaction^c^	1.9	0.660	
111^b^	Takes over a simple message^c^	7.8^d^	0.383	
	**2 Negative emotionality**			0.80
81	Is stubborn, sullen, or irritable	5.5	0.790	
82	Changes mood suddenly	2.2	0.755	
43	Seems unhappy without clear reason	2.3^d^	0.715	
88	Is uncooperative	2.5	0.712	
29	Is easily upset	4.6	0.679	
13	Cries a lot	1.9	0.675	
66	Screams a lot	5.2	0.649	
69	Will not share toys or other things	1.4	0.574	
42	Hurts animals or persons (unintentionally)	0.9	0.532	
40	Bites, hits, or kicks others	2.3	0.519	
11	Wants help constantly	1.3	0.494	
91	Is extremely loud	1.9	0.492	
62	Refuses to play active games him/herself	5.8^d^	0.402	
34	Is accident prone	3.5	0.368	
32	Is afraid of certain animals, situations, or places	11.1	0.304	
	**3 Attention-deficit/hyperactivity problems**			0.66
6	Cannot sit still; is restless or hyperactive	5.7	0.733	
5	Cannot concentrate or pay attention for long	3.6	0.658	
138^b^	Sits still for five minutes during reading together	1.8	0.483	
	**4 Deviant play behaviour**			0.63
106^b^	Plays in various ways	0.8	0.682	
103^b^	Plays with different toys/objects	1.6	0.607	
105^b^	Shows interest in new objects/persons	0.7	0.486	
	**5 Demanding behaviour**			0.58
96	Wants a lot of attention	10.8	0.589	
16	Demands must be met immediately	15.1	0.579	
97	Is fussy, whiny	5.3	0.578	
8	Cannot stand waiting; wants everything now	13.9	0.532	
44	Has angry moods	6.6	0.437	
59	Quickly shifts from one activity to another	10.1	0.365	
	**6 Deviant affective behaviour**			0.50
114^b^	Uses gestures appropriately to express him/herself	5.3^d^	0.436	
115^b^	Shows clear facial expressions	5.7^d^	0.434	
109^b^	Emotions are understandable	8.3^d^	0.428	
112^b^	Reacts normally to sensory stimuli	2.0^d^	0.427	
	**7 Communication and interaction problems**			0.78
136^b^	Directs social smile to parents and others	4.1^d^	0.783	
135^b^	Follows glance of parents^c^	2.7	0.718	
129^b^	Uses sounds or words to get attention or help^c^	2.5	0.662	
123^b^	Clings on when he/she wants to be held	1.0	0.660	
130^b^	Shows interest in other children/adults	7.8^d^	0.646	
125^b^	Utters sounds of joy	1.1	0.642	
127^b^	Gives or shows something to somebody	0.8	0.616	
139^b^	Likes to play games with others	3.7^d^	0.611	
142^b^	Reacts when being spoken to	3.7^d^	0.526	
137^b^	Pays attention when being spoken to	0.7	0.509	
121^b^	Asks attention when being alone	6.0	0.531	
141^b^	Reacts when name is called	3.7^d^	0.511	
132^b^	Enjoys learning new things	7.1^d^	0.499	
120^b^	Shows that he/she distinguishes parents from others	3.8^d^	0.486	
133^b^	Likes cuddling	8.7^d^	0.485	
118^b^	Makes eye contact easily	7.9^d^	0.471	
104^b^	Reaches when he/she wants to be held	7.6	0.456	
134^b^	Stops wailing when being spoken to	1.7	0.446	
128^b^	Uses objects for imaginative play	1.0	0.445	
122^b^	Wants to do things him/herself	7.6^d^	0.420	
108^b^	Imitates complex tasks^c^	6.3	0.361	
	**8 Sleep problems**			0.67
22	Cannot sleep alone	6.5	0.815	
38	Finds it difficult to fall asleep	5.7	0.762	

^a^Total number of items was 62. Only items with factor loading ≥ 0.30 were included (58). Other items were omitted (4): 24 Does not eat well; 102 Points at things to show; 110 Keeps on trying; 124 Repeats stereotypic movements.

^b^Items were reversely coded.

^c^Adjusted or alternative items.

^d^Proportions of children with score ‘1’ and ‘2’ were combined and considered as deviant (<limit of 10%); in all other cases only a score of 2 represented deviant behaviour.

### Statistical approach

To interpret all items similarly, some items were reversely coded. A score of 0 meant that a child showed normal behaviour; a score of 1 or 2 implied that a child lacked competences or experienced problems to a mild or severe degree. The items were considered as ordinal variables, had the same weight, and were of equal importance. Maximally six missing values on each questionnaire were allowed, and these values were imputed by means of single imputation using expectation maximization techniques [28].

Previous results of the exploratory factor analysis (EFA) of USQ data in the large population-based sample (*N* = 6,330) at T1 were considered valid and reliable [20]. Therefore, they served as starting point for the analyses of the USQ data in the smaller sample (*N* = 4,237) at T2. Although the number of items was reduced, from 74 in the USQ to 62 in the SBQ, roughly the same factor solution was used. The factors at T2 were determined by EFA, which was executed using the weighted least squares means and variance-adjusted (WLSMV) estimator. The optimal number of factors was based on the bend in the scree-plot, a small root mean square residual (RMSR), no or a few negative estimated residual variances (ERVs), and an intelligible interpretation of the factors. Internal consistency and variance explained were computed to establish reliability. Correlations between factors of the SBQ were calculated to get insight into their interrelatedness.

To examine the existence of unobserved population heterogeneity, factor mixture models (FMM) [10] were applied at T1 and T2 separately. FMM classifies individuals in homogeneous groups (latent classes), just as in cluster analysis and latent class analysis (LCA). In a standard LCA, variables are considered to be conditionally independent within each class. In contrast, in FMM it is assumed that variables within each class can be combined using a common factor analysis. Both factor division and class membership are latent, i.e., it is neither directly known how a subject scores on the underlying factors, nor to which class a subject belongs, but this information can be gathered later on. The FMMs were performed using the maximum likelihood estimator with robust standard errors (MLR). The best fitting model was identified on the basis of low (sample-size adjusted) Bayesian information criterion ((SSA) BIC) values, significant *p*-values like Vuong-Lo-Mendell-Rubin likelihood ratio test (VLMR LRT) and Lo-Mendell-Rubin adjusted likelihood ratio test (LMR adj. LRT), high entropies, and clear interpretations of the classes/profiles [29]. Children could only be admitted to one class. The distributions of respondent, sex, nationality, and SES were analysed across classes by means of crosstabs, Chi-square tests, and adjusted residuals. Differences in mean age per class were evaluated with one-way ANOVA and Bonferroni corrected post hoc tests.

After FMM, weighted factor scores were computed by dividing the obtained factor sum score by the maximum factor sum score, first for each individual and later for each class as a whole. These continuous factor scores with values between 0 and 1 enable the comparison of classes on several factors within one instrument at one moment (either USQ or SBQ), and the comparison of classes on similar factors between two instruments at different time points (both USQ and SBQ). The overall size and significance of differences between classes were determined with one-way ANOVA and Bonferroni corrected post hoc tests. More precise differentiations between the classes were given by Cohen’s *d* effect sizes. These data provided information about qualitative and quantitative differences in weighted factor scores. Analyses were repeated with sex as covariate to determine whether it was necessary to distinguish between boys and girls.

To gain insight into the stability of behavioural problems over time, we used a variable- based and a person-based approach. To establish the specific stability of problem domains over time, we made a matrix with correlations between the factors of the USQ (T1) and the SBQ (T2). To determine the continuity of behavioural and developmental profiles over the 2-year period, a crosstab with percentages and adjusted residuals (*M* = 0 and *SD* = 1) was produced. Relevant transitions between classes from USQ to SBQ were depicted in a transition model. Next, we computed a dummy variable (0 = drop-outs; 1 = follow-ups) and tested for selective attrition per nationality, SES, sex and class through crosstabs and Chi-square tests. Thereafter, we analysed per class whether the children who completed the follow-up were representative of the whole class. Independent samples T-tests were used to determine whether weighted factor scores for drop-outs and follow-ups differed significantly. Analyses were carried out with Mplus version 4.1 [30] or SPSS 17.0 [31].

## Results

**Figure 1:**
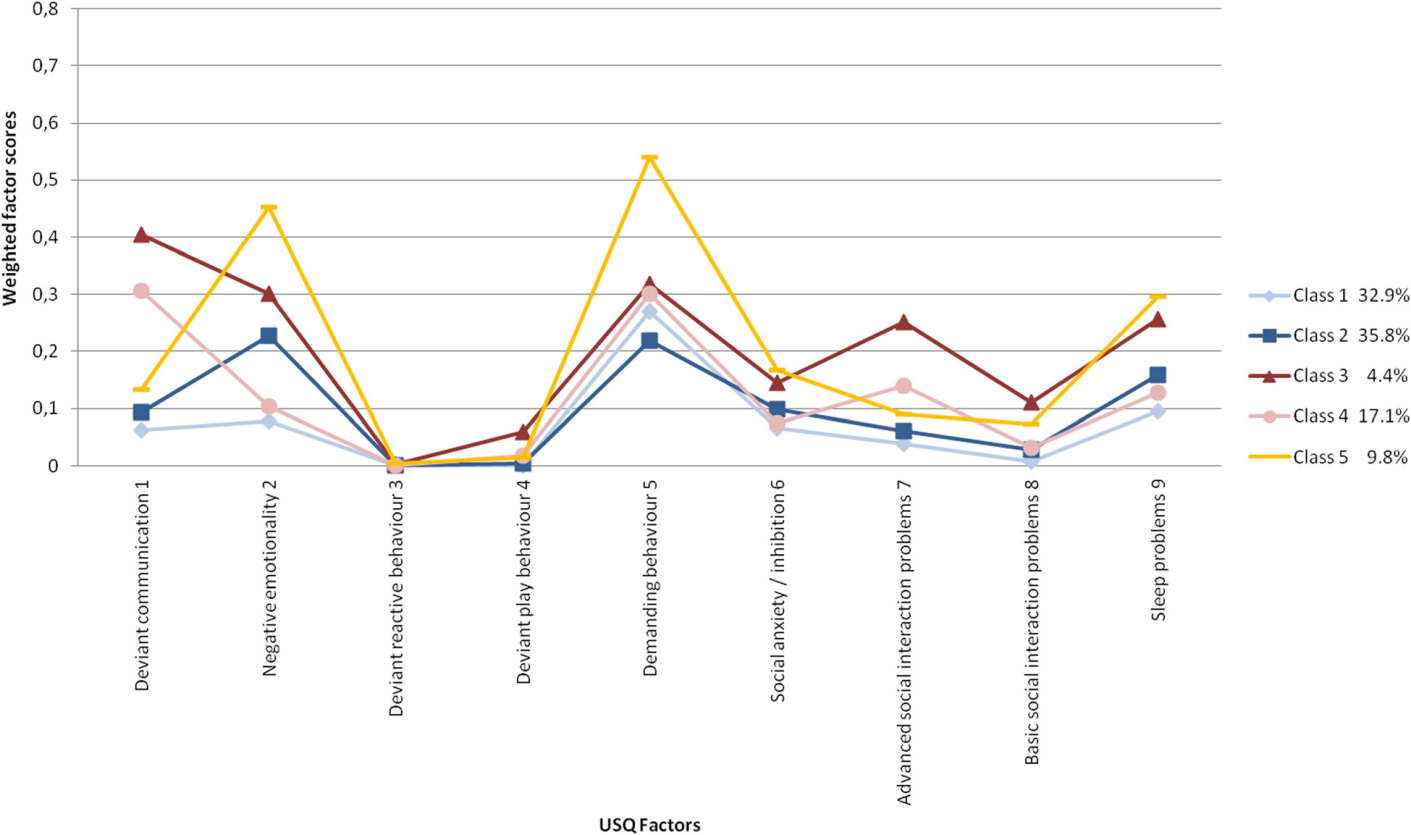
**USQ weighted factor scores per class at T1 (*N*** **= 4,237).**

Previous analyses of USQ data at T1 (*N* = 6,330) revealed nine factors and five classes/profiles of which three were indicative of increased problems [20]. Information regarding factor solution, class division, and profiles for the sample with 4,237 children for whom data were available at T2 are presented in Tables 1, 2, 3, 4 and 5, and Figure 1.

**Table 3:** **Summary of results of factor mixture modelling of USQ and SBQ (*N*** **= 4,237)**

	**Classes**	**General tests of model fit**			**Technical 11 output**	
	**Number**	**Entropy**	**BIC**	**SSA BIC**	**VLMR LRT**	**LMR adj. LRT**
					***p*-value**	***p*-value**
**USQ (T1)**	4	0.823	275,706.768	274,928.260	0.0000	0.0000
	5	0.821	274,767.281	273,890.269	0.0007	0.0007
	6	0.806	274,233.404	273,257.887	1.0000	1.0000
**SBQ (T2)**	4	0.873	271,474.396	270,759.440	0.0000	0.0000
	5	0.862	269,513.215	268,744.240	0.0037	0.0038
	6	0.853	268,265.297	267,442.303	0.0039	0.0040
	7	0.861	267,610.793	266,733.781	0.1297	0.1311

*Note.* Entropy indicates classification accuracy. *BIC* Bayesian Information Criterion, *SSA BIC* Sample-Size Adjusted Bayesian Information Criterion, *VLMR LRT* Vuong-Lo-Mendell-Rubin Likelihood Ratio Test, *LMR adj. LRT* Lo-Mendell-Rubin Adjusted Likelihood Ratio Test.

**Table 4:** **Prevalence estimates and distribution of age, sex, nationality, and SES for five-class-model of USQ and SBQ (*N =*** **4,237)**

**USQ (T1)**	**Class 1 *n *** **(%)**	**Class 2 *n *** **(%)**	**Class 3 *n *** **(%)**	**Class 4 *n *** **(%)**	**Class 5 *n *** **(%)**	**Total *n *** **(%)**	***df***	***F*; *p *** **(age) *χ***^**2**^**; *p *** **(others)**
**Age** (child) (*M, SD)*	14.71 (0.65)	14.74 (0.76)	14.68 (0.55)	14.56 (0.56)^*^	14.72 (0.64)	14.69 (0.68)	4, 4216	9.08; < 0.001
**Sex** (child)							4	21.60; < 0.001
Boys	651 (46.6)^b^	793 (52.3)	98 (53.0)	401 (55.2)	233 (56.3)	2,176 (51.4)		
Girls	745 (53.4)^a^	723 (47.7)	87 (47.0)	325 (44.8)	181 (43.7)	2,061 (48.6)		
**Nationality** (child)							8	60.71; < 0.001
Dutch	1,354 (97.0)^a^	1,432 (94.5)	160 (86.5)^b^	692 (95.3)	375 (90.6)^b^	4,013 (94.7)		
Non-Dutch	39 (2.8)^b^	77 (5.1)	25 (13.5)^a^	32 (4.4)	38 (9.2)^a^	211 (5.0)		
**SES** (parents)							12	130.88; < 0.001
Low	111 (8.0)^b^	144 (9.5)	40 (21.6)^a^	100 (13.8)	83 (20.0)^a^	478 (11.3)		
Moderate	486 (34.8)^b^	622 (41.0)	86 (46.5)	291 (40.1)	184 (44.4)	1,669 (39.4)		
High	795 (56.9)^a^	741 (48.9)	58 (31.4)^b^	332 (45.7)	145 (35.0)^b^	2,071 (48.9)		
**Prevalence**	1,396 (32.9)	1,516 (35.8)	185 (4.4)	726 (17.1)	414 (9.8)	4,237 (100.0)		
**SBQ (T2)**	**Class 1 *n *** **(%)**	**Class 2 *n *** **(%)**	**Class 3 *n *** **(%)**	**Class 4 *n *** **(%)**	**Class 5 *n *** **(%)**	**Total *n *** **(%)**	***df***	***F*; *p *** **(age) *χ***^**2**^**; *p *** **(others)**
**Age** (child) (*M, SD)*	36.71 (2.59)	36.44 (2.62)^*^	36.66 (2.69)	36.91 (2.64)	36.77 (2.66)	36.64 (2.63)	4, 4220	4.62; 0.001
**Sex** (child)							4	16.76; 0.002
Boys	642 (48.3)	827 (53.2)	225 (57.1)	351 (48.3)	131 (56.0)	2,176 (51.4)		
Girls	686 (51.7)	728 (46.8)	169 (42.9)	375 (51.7)	103 (44.0)	2,061 (48.6)		
**Nationality** (child)							8	72.53; < 0.001
Dutch	1,282 (96.5)^a^	1,478 (95.0)	354 (89.8)^b^	696 (95.9)	203 (86.8)^b^	4,013 (94.7)		
Non-Dutch	40 (3.0)^b^	71 (4.6)	40 (10.2)^a^	29 (4.0)	31 (13.2)^a^	211 (5.0)		
**SES** (parents)							12	247.67; < 0.001
Low	94 (7.1)^b^	140 (9.0)^b^	93 (23.6)^a^	97 (13.4)	54 (23.1)^a^	478 (11.3)		
Moderate	425 (32.0)^b^	615 (39.5)	180 (45.7)	341 (47.0)^a^	108 (46.2)	1,669 (39.4)		
High	803 (60.5)^a^	791 (50.9)	118 (29.9)^b^	287 (39.5)^b^	72 (30.8)^b^	2,071 (48.9)		
**Prevalence**	1,328 (31.4)	1,555 (36.7)	394 (9.3)	726 (17.1)	234 (5.5)	4,237 (100.0)		

*Note.* Percentages of demographic characteristics are given for each individual class, so that the total amounts to (approximately) 100 vertically. However, percentages regarding prevalence add up to 100 horizontally.

*USQ: Children in class 4 were significantly younger than children in classes 1, 2, and 5 (*p* < 0.001).

SBQ: Children in class 2 were significantly younger than children in class 4 (*p* < 0.001).

Adjusted residuals revealed significant differences in percentages of children in classes on variables sex, nationality, and SES (*p* < 0.001).

^*a*^Percentage was significantly *higher* than the overall average percentage.

^*b*^Percentage was significantly *lower* than the overall average percentage.

**Table 5:** **Weighted factor scores (wfs; proportions) and Cohen's *d *** **values of USQ and SBQ factors (*N*** **= 4,237)**

**Factors USQ (T1)**	**Class 1**		**Class 2**		**Class 3**		**Class 4**		**Class 5**		***F*** **(4, 4232); *p***^**#**^
	**32.9%**		**35.8%**		**4.4%**		**17.1%**		**9.8%**		
	**wfs**	***d***	**wfs**	***d***	**wfs**	***d***	**wfs**	***d***	**wfs**	***d***	
1 Deviant communication	0.06	*0.00*	0.09	*0.36*	0.40	2.49^**^	0.31	2.12^**^	0.13	*0.69*	962.86; < 0.001
2 Negative emotionality	0.08	*0.00*	0.23	2.43^**^	0.30	2.80^**^	0.10	*0.43*	0.45	4.67^**^	2,904.45; < 0.001
3 Deviant reactive behaviour	0.00	*0.00*	0.00	*-0.04*	0.00	*0.08*	0.00	*0.02*	0.00	*0.10*	3.68; < 0.001
4 Deviant play behaviour	0.00	*0.00*	0.00	*0.13*	0.06	*0.57*	0.02	*0.30*	0.01	*0.29*	55.35; < 0.001
5 Demanding behaviour	0.27	*0.00*	0.22	*-0.27*	0.32	*0.23*	0.30	*0.20*	0.54	1.08^*^	203.11; < 0.001
6 Social anxiety/inhibition	0.07	*0.00*	0.10	*0.25*	0.14	*0.52*	0.07	*0.07*	0.17	*0.69*	53.27; < 0.001
7 Advanced social interaction problems	0.04	*0.00*	0.06	*0.40*	0.25	2.04^**^	0.14	1.30^*^	0.09	0.81^*^	527.95; < 0.001
8 Basic social interaction problems	0.01	*0.00*	0.03	*0.30*	0.11	*0.77*	0.03	*0.34*	0.07	*0.67*	88.53; < 0.001
9 Sleep problems	0.10	*0.00*	0.16	*0.38*	0.26	0.90^*^	0.13	*0.22*	0.30	1.14^*^	138.49; < 0.001
**Factors SBQ (T2)**	**Class 1**		**Class 2**		**Class 3**		**Class 4**		**Class 5**		***F*** **(4, 4232); *p***^**#**^
	**31.4%**		**36.7%**		**9.3%**		**17.1%**		**5.5%**		
	**wfs**	***d***	**wfs**	***d***	**wfs**	***d***	**wfs**	***d***	**wfs**	***d***	
1 Language problems	0.03	*0.00*	0.04	*0.19*	0.29	1.39^**^	0.10	*0.62*	0.13	*0.69*	351.52; < 0.001
2 Negative emotionality	0.06	*0.00*	0.22	2.39^**^	0.26	2.50^**^	0.08	*0.44*	0.44	4.03^**^	2,087.01; < 0.001
3 Attention-deficit/hyperactivity problems	0.06	*0.00*	0.21	0.97^*^	0.34	1.59^**^	0.11	*0.37*	0.47	2.00^**^	451,86; < 0.001
4 Deviant play behaviour	0.01	*0.00*	0.03	*0.29*	0.21	1.27^*^	0.13	0.95^*^	0.10	*0.77*	313.50; < 0.001
5 Demanding behaviour	0.33	*0.00*	0.19	-0.99^*^	0.24	*-0.63*	0.31	*-0.17*	0.71	2.42^**^	680.53; < 0.001
6 Deviant affective behaviour	0.00	*0.00*	0.01	*0.27*	0.13	1.07^*^	0.04	*0.61*	0.07	0.81^*^	263.49; < 0.001
7 Communication and interaction problems	0.04	*0.00*	0.06	*0.67*	0.24	2.41^**^	0.15	2.20^**^	0.13	1.32^**^	1,188.02; < 0.001
8 Sleep problems	0.10	*0.00*	0.21	*0.44*	0.24	*0.53*	0.10	*-0.03*	0.34	0.84^*^	86.07; < 0.001

#The weighted factor score is the obtained factor sum score divided by the maximum factor sum score, and has a value between 0 and 1. Higher scores indicated that children lacked more competences or showed more problems. Most differences in weighted factor scores between classes were significant (*p* < 0.001). However, the following contrasts were *not* significantly different: **USQ**: *factor 3* classes 1, 2, 3, 4, and 5; *factor 4* classes 1 and 2, classes 2 and 5, classes 4 and 5; *factor 5* classes 1, 3, and 4; *factor 6* classes 1, 2, and 4, classes 3 and 5; *factor 8* classes 2 and 4; *factor 9* classes 3 and 5. **SBQ**: *factor 1* classes 1 and 2, classes 4 and 5; *factor 4* classes 4 and 5; *factor 5* classes 1 and 4; *factor 6* classes 1 and 2; *factor 8* classes 1 and 4, classes 2 and 3.

Cohen’s *d*: comparison between the weighted factor score of class 1 versus classes 2, 3, 4, and 5; * large effect size (*d* ≥ 0.80); ** very large effect size (*d* ≥ 1.30).

### Factors at 36–37 months

The structure of behaviour was examined by entering all 62 items in EFA, but only those (58) with factor loadings ≥ 0.30 were used. Each item was assigned to the factor on which it had the highest loading; cross-loadings were neglected. All meaningful factors (with an eigenvalue ≥ 1.40) were included. A solution with eight factors seemed to be best, because the RMSR was acceptably small (0.0339) and there were no negative ERVs. The factors were termed language problems, negative emotionality, attention-deficit/hyperactivity problems, deviant play behaviour, demanding behaviour, deviant affective behaviour, communication and interaction problems, and sleep problems (Table 2).

The internal consistency (Cronbach’s alpha) of the separate factors varied from 0.50 to 0.80 (Table 2). The factors with a poor or questionable internal consistency mostly contained a small number of items and/or items that assess rare or rather extreme behaviour. The percentages of variance explained amounted to 37.8%. When all 58 items were considered together, internal consistency was good (***α*** = 0.84) and variance explained was 72.5%. Interrelationships between the eight factors of the SBQ were computed, resulting in 28 correlations: 10 were negligible (*r* < 0.10), 13 were small (*r* = 0.10 - 0.30), and 5 were moderate (*r* = 0.30 - 0.50). Communication and interaction problems correlated with language problems (*r* = 0.42) and deviant play behaviour (*r* = 0.36); negative emotionality correlated with language problems (*r* = 0.36), ADHD problems (*r* = 0.33), and sleep problems (*r* = 0.33).

The factor solutions at 14–15 and 36–37 months were comparable, but in toddlerhood there was one factor less, the content of the factors was slightly different, and the factor ‘language problems’ was more prominent than in infancy.

### Classes and profiles at 36–37 months

FMM identified specific behavioural and developmental profiles as well as the accompanying proportions of children. Table 3 shows the measures of fit and accuracy. The (SSA)BIC continued to decline up till seven classes. However, a 7-class solution was not better in LRT values than a 6-class solution, but the 6-class solution showed improvement over the 5-class solution. Based on these criteria we should have chosen the 6-class solution. However, this significant difference was mainly due to the large population size. The 6-class solution showed an extra normal group in comparison to the 5-class solution. In both solutions, the total number of children with typical behaviour was comparable. The corresponding profiles were very similar and showed only small differences in severity. Because the groups reflecting normal behaviour were of minor clinical importance, and because an equal number of analogous groups at T1 and T2 will lead to a clearer transition model, the 5-class solution was adopted.

**Figure 2:**
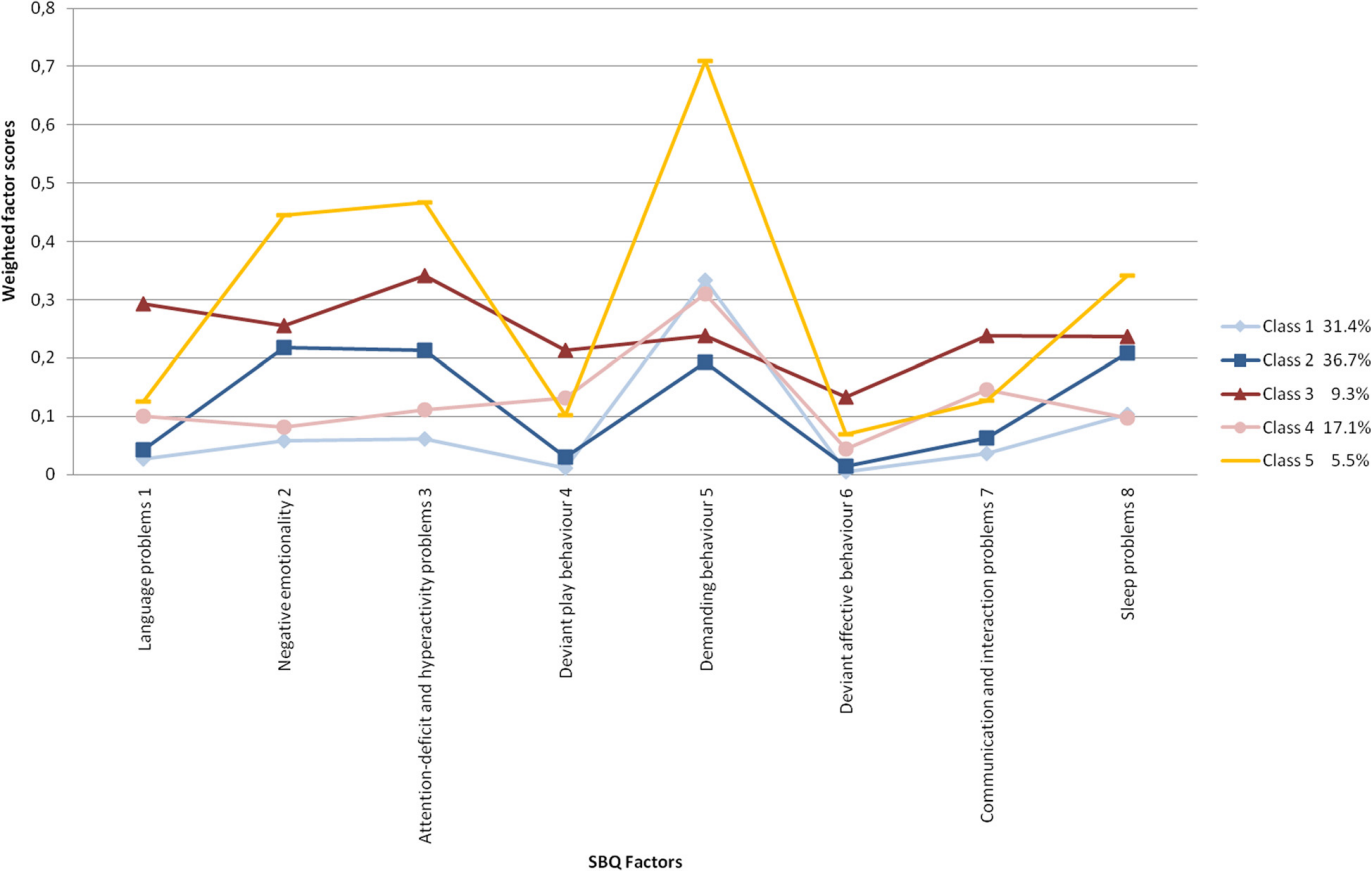
**SBQ weighted factor scores per class at T2 (*N*** **= 4,237).**

A total of 31.4% of the children belonged to class 1, 36.7% to class 2, 9.3% to class 3, 17.1% to class 4, and 5.5% to class 5. There were no significant differences in respondent (same or different) between the classes, neither at T1 (***χ***^2^ = 0.086) nor at T2 (***χ***^2^ = 0.280). Age differed significantly between the classes (*p* < 0.001), with children in class 2 being 10 days younger (*M* = 36.44 months) than the children in the other classes, who were about the same age (*M* = 36.77 months). The distribution of boys and girls in the five classes did not differ significantly from the overall mean distribution. Class 1 consisted of many Dutch children (96.5%) and children from families with a high SES (60.5%), compared to the total mean. In contrast, classes 3 and 5 contained a higher proportion of children with a non-Dutch nationality (10.2% and 13.2% respectively) than average. In classes 3, 4, and 5, children from high SES backgrounds were underrepresented (29.9%, 39.5%, and 30.8% respectively). See Table 4.Results of FMM are presented in a line chart with continuous weighted factor scores for each class separately (Figure 2). No other parameters were estimated across classes, thus the class division was solely based on these factor scores. A higher score indicated that toddlers lacked more competences or showed more problems. Globally, three groups could be distinguished, namely one group (classes 1, 2, and 4) consisting of relatively normal children, one group (class 3) consisting of children with communication and/or social interaction problems, and one group (class 5) consisting of children with negative and demanding behaviour. The five classes and profiles showed both quantitative and qualitative differences. Class 1 had relatively low scores on all factors and was considered the reference group with typical children. Class 2 was normal in most respects, but showed mild negative behaviour. Scores on negative emotionality and ADHD problems were higher, but scores on demanding behaviour were lower than those of class 1. Class 4 had mild communication and interaction problems and showed deviant play behaviour, but was otherwise normal. Class 3 was characterized by moderate scores on negative emotionality and ADHD problems, and high scores on language problems and factors involving communication and social interaction, findings suggestive of a wide variety of developmental problems. Class 5 was especially characterized by high scores on negative emotionality, ADHD problems, and demanding behaviour. These children seemed to be at comparatively high risk for externalizing problems.

For each separate factor, the continuous weighted factor scores of all five classes were compared with each other. One-way ANOVA and Bonferroni corrected post hoc tests revealed that all but seven differences were significant (*p* < 0.001). Several (very) large effect sizes, expressed in Cohen’s *d*, were found between class 1 on the one hand and classes 2, 3, 4, and 5 on the other. Classes 3 and 5 stood out because the weighted factor scores of six of the eight factors were significantly higher than those of class 1. See also Table 5. Analyses were repeated with inclusion of covariate sex in the model, which did not reveal significantly different levels of problems between boys and girls. There were no other covariance parameters included.

### Longitudinal stability of factors and classes

Item scores with values 0, 1, and 2 were used to compute weighted factor scores. Obtained factor sum scores were divided by maximum factor sum scores, what resulted in continuous weighted factor scores with values between 0 and 1.

Most correlations between the factors at age 14–15 months and at age 36–37 months were small, but significant (*p* ≤ 0.001). See Table 6. The highest correlations were found between factors with overlapping or similar items: deviant communication (USQ) and language problems (SBQ) (*r* = 0.35); negative emotionality (USQ) and negative emotionality and attention-deficit/hyperactivity problems (SBQ) (*r* = 0.44 and *r* = 0.32, respectively); advanced social interaction problems (USQ) and communication and interaction problems (SBQ) (*r* = 0.34); sleep problems (USQ) and sleep problems (SBQ) (*r* = 0.31).

**Table 6:** **Pearson correlation matrix of USQ and SBQ factors (*N*** **= 4,237)**

**Factors USQ (T1)**	**Factors SBQ (T2)**							
	**1 Language problems**	**2 Negative emotionality**	**3 ADHD- problems**	**4 Deviant play behaviour**	**5 Demanding behaviour**	**6 Deviant affective behaviour**	**7 Com. interact. problems**	**8 Sleep problems**
1 Deviant communication	0.35^*^	0.09^*^	0.10^*^	0.15^*^	0.05	0.17^*^	0.25^*^	-0.00
2 Negative emotionality	0.07^*^	0.44^*^	0.32^*^	0.12^*^	0.07^*^	0.11^*^	0.14^*^	0.15^*^
3 Deviant reactive behaviour	0.00	0.02	-0.01	0.05^*^	0.00	0.03	0.02	0.01
4 Deviant play behaviour	0.12^*^	0.04	0.08^*^	0.11^*^	0.04	0.10^*^	0.14^*^	-0.02
5 Demanding behaviour	0.04	0.10^*^	0.10^*^	0.03	0.19^*^	0.04	0.05^*^	0.04
6 Social anxiety/inhibition	0.05^*^	0.12^*^	0.05^*^	0.06^*^	0.03	0.05^*^	0.04	0.07^*^
7 Advanced social interaction problems	0.23^*^	0.09^*^	0.13^*^	0.23^*^	0.03	0.19^*^	0.34^*^	0.02
8 Basic social interaction problems	0.14^*^	0.19^*^	0.20^*^	0.15^*^	0.06^*^	0.13^*^	0.22^*^	0.09^*^
9 Sleep problems	0.06^*^	0.17^*^	0.12^*^	0.09^*^	0.06^*^	0.09^*^	0.13^*^	**0.31**

*Most correlations (*r)* were small, but significant (*p* ≤ 0.001). The **highest correlations** were found between deviant communication (USQ) and language problems (SBQ); negative emotionality (USQ) and negative emotionality / attention-deficit/hyperactivity problems (SBQ); advanced social interaction problems (USQ) and communication and interaction problems (SBQ); sleep problems (USQ) and sleep problems (SBQ).

At age 36–37 months, the profiles showed more variation in the type of behaviour and behaviour seemed to be more crystallized, especially in classes 3 and 5, compared to the profiles at age 14–15 months. The proportion of children with normal behaviour (class 1) was similar at T1 and T2 (32.9% versus 31.4%), but there were more children with mild problems (classes 2 and 4 53.8%) at T2 than at T1 (class 2 35.8%). Some children had moderate problems (class 4 17.1%) at T1, but this type of problem was no longer seen at T2. The proportion of children with severe problems was about the same at T1 and T2 (14.2% versus 14.8%). The proportion of children with communication and interaction problems (class 3) or with negative and demanding behaviour (class 5) was reversed at T1 (4.4% and 9.8%, respectively) and T2 (9.3% and 5.5%, respectively).

**Figure 3:**
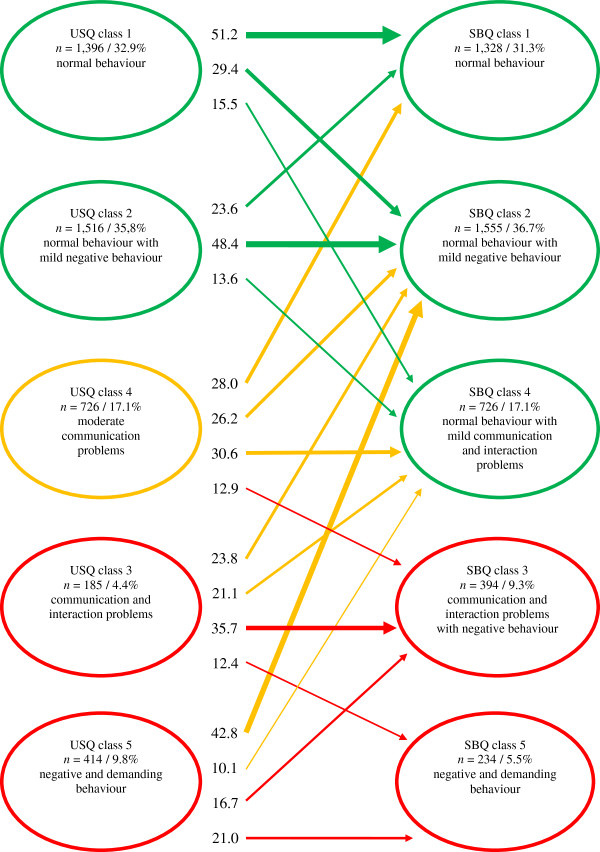
**Transition model for USQ 5 classes (T1) and SBQ 5 classes (T2) (*N*** **= 4,237).***Note.* The order of class 3 and class 4 is changed for reasons of clarity. Transitions are given in percentages per class. Frames: green = (near) normal behaviour; orange = moderate problems; red = severe problems. Arrows: green = (near) normal at T1 and T2; orange = problematic at T1, but (near) normal at T2; red = problematic at T1 and T2.

Crosstabs were calculated to examine significant changes in behavioural profiles over time. Transitions that affected more than 10% of the children and that had adjusted residuals of 2 or higher (*p* = 0.05) (i.e. difference between observed and expected frequency divided by an estimate of its standard deviation) are reported (Figure 3). There were five main findings. First, children who showed normal or near normal behaviour (classes 1 and 2) at T1 had similar behaviour (classes 1, 2, and 4) at T2 (green arrows), thus children with near normal behaviour in infancy did not develop deviant behaviour in toddlerhood. Second, the majority (85%) of children with moderate communication problems (class 4) at T1 showed normal behaviour (or with only mild problems) (classes 1, 2, and 4) at T2 (orange arrows); however, some children (15%) had severe problems at T2, especially communication and interaction problems (class 3) (red arrow). Third, children with severe problems (classes 3 and 5) at T1 did not have completely normal behaviour (class 1) at T2; however, the behaviour of a substantial proportion of children with severe problems (44.9% of class 3 and 52.9% of class 5) improved to near normal behaviour (classes 2 and 4) (orange arrows). Fourth, negative and demanding behaviour tended to improve over time more (62.3% of the children from class 5 at T1 shifted to classes 1, 2, and 4 at T2) than did communication and interaction problems (51.9% of the children from class 3 at T1 shifted to classes 1, 2, and 4 at T2). Fifth, homotypic continuity occurred more often than heterotypic continuity in children with communication and interaction problems (35.7% homotypic versus 12.4% heterotypic (from class 3 at T1 to class 3 and 5 at T2, respectively)) than in children with negative and demanding behaviour (21.0% homotypic versus 16.7% heterotypic (from class 5 at T1 to classes 5 and 3 at T2, respectively)) (red arrows).

### Representativeness of follow-up sample

At T2 (*N* = 4,237) the sample was smaller than at T1 (*N* = 6,330); the follow-up rate was 66.9%. We checked for selective attrition and found follow-up rates to be different by demographic data (*p* < 0.001). The follow-up rate was lower for children with a Moroccan or Turkish nationality than for Dutch children (33.8% versus 69.9%). The follow-up rates of children with European and other nationalities were in between. Regarding the SES as well as the level of education and occupation can be concluded that the follow-up rate was lowest for the children of parents with a low level (about 60%) and highest for the parents with a high level (about 70%). The follow-up rate for boys and girls was 67.9% and 68.2% respectively (*p* = 0.78). The follow-up rates for children in the different behavioural classes were: class 1 70.6%, class 2 66.7%, class 3 50.8%, class 4 69.9%, and class 5 60.5% (*p* < 0.001). As expected, the follow-up rate was lowest for children with communication and interaction problems (class 3) and for children with negative and demanding behaviour (class 5). Independent samples T-tests comparing the weighted factor scores of children in class 3 at T1 revealed that drop-outs scored significantly higher on social anxiety/inhibition (*p* = 0.002), advanced social interaction problems (*p* < 0.001), and sleep problems (*p* = 0.002) than follow-ups. The higher symptom severity at T1 of drop-outs suggests that the proportion of children with persistent communication and interaction problems at T2 was underestimated. At T1, children of class 5 who dropped out had slightly more advanced social interaction problems (*p* = 0.02) and sleep problems (*p* = 0.03) than those who completed the follow-up. Differences on other factors were not significant, in particular not on negative emotionality (*p* = 0.71), which factor included items most characteristic for negative and demanding behaviour, suggesting that overall follow-ups at T2 were representative of class 5.

## Discussion

We explored the course of a small number of parent-reported problem behaviours in a large longitudinal population-based sample of Dutch children from age 14–15 months (T1) to age 36–37 months (T2). While negative emotionality, demanding behaviour, deviant play behaviour, and sleep problems tended to group similarly at both time points, deviant communication, social anxiety/inhibition, as well as basic and advanced social interaction problems at T1 were combined in one factor termed communication and interaction problems at T2. As expected, language problems was identified as a new factor in toddlerhood. The changes are partly due to a decrease in the number of participants (from 6,330 infants at T1 to 4,237 toddlers at T2) and a reduction in the number of items (from 74 items in USQ to 62 items in SBQ). These can be considered as measurement effects. Besides, developmental effects also played a role.

The proportion of children with normal behaviour or mild negative behaviour was similar at both ages (nearly 70%), and no children with normal behaviour at 14–15 months had deviant behaviour at 36–37 months. Most infants (84.8%) with moderate communication problems showed near normal behaviour in toddlerhood. However, communication and interaction problems as well as negative and demanding behaviour in infancy were strong predictors of mild to severe problems in toddlerhood, as no infants with these problems showed completely normal behaviour as toddlers. However, 62.3% of infants with negative and demanding behaviour showed behavioural improvement in toddlerhood, compared with 51.9% of infants with communication and interaction problems. In addition, homotypic continuity was less pronounced for negative and demanding behaviour (21.0% homotypic versus 16.7% heterotypic) than for communication and interaction problems (35.7% homotypic versus 12.4% heterotypic). The proportion of children with negative and demanding behaviour decreased, but their behaviour problems became more severe. In contrast, communication and interaction problems affected more children and were often seen in combination with language problems and moderate negative behaviour. Thus, negative and demanding behaviour would appear to be transient more often and a less specific predictor of problems in toddlerhood than communication and interaction problems.

Our research has several scientific and clinical implications. Forty percent of the toddlers with communication and interaction problems had these problems as infants, a finding consistent with the results of a large screening study of early symptoms of ASD in the general population [26,27]. At the same time, however, about 40% of the children with communication and interaction problems in toddlerhood were not identified and reported correctly by their parents in infancy, possibly because the symptoms were less obvious and severe, or because the parents were not able or willing to recognize them properly. Therefore, it is essential to improve the methods and instruments for the identification of more subtle symptoms of infants, in addition to a repeated screening and evaluation of communication and interaction problems of toddlers. Our data regarding communication and interaction problems are not very fine grained and do not include information about regressive behaviours. Consequently, they suggest only two patterns of ASD onset: a very early onset and a somewhat later onset. However, these different trajectories partly correspond with the results of smaller but more detailed studies of the early development of clinically referred children with communication and interaction problems, i.e. children with ASD. Ozonoff et al. [32] identified three patterns of onset of ASD: early onset, regression, and plateau. More research is needed, bearing in mind that symptom emergence can be considered as a continuum of many phenotypes containing different characteristics and varying in severity [5,32,33].

Although negative and demanding behaviour may be transient in many children, it may be persistent in some others and can lead to serious child psychiatric disorders, such as ADHD. According to Sonuga-Barke and Halperin [4], the onset of ADHD is best described through syndrome trajectories that allow fluctuations over time (emergence, persistence, decrease, increase) depending on the child’s chronological and developmental age. Willoughby, Pek, and Greenberg [34] distinguished three main patterns of ADHD symptoms, namely, consistently low, remittent, and persistently high, in a population-based sample of preschoolers. In our research, behaviour improved in a substantial proportion of children (at least 40%). Though, it remains to be seen whether this improvement is permanent, or whether negative and demanding behaviour re-emerges in childhood. However, in about 20% of the children, externalizing problems were present in both infancy and toddlerhood, and may remain throughout childhood and later adolescence. These children may form a group with persistent externalizing problems, who may benefit from early intervention.

Thus, various trajectories of ASD on the one hand and ADHD on the other hand can be distinguished. However, severe communication and social interaction problems may also occur in combination with persistent externalizing behaviour. Both conditions may reinforce each other, may complicate development, and may influence functioning [35]. ASD and ADHD may share genetic and environmental risk factors, developmental pathways, as well as cognitive and neural mechanisms [36,37]. It has even been suggested that the two conditions represent different manifestations of a common underlying disorder [38]. Our findings provide a context to examine the shared and unique behavioural precursors of ASD and ADHD.

Albeit the study was carried out in a large population-based sample, it had some limitations. The T2 sample comprised 4,237 children, only 34.5% of the total sample (*N =* 12,297). The T2 sample mainly consisted of children of Dutch parents with a relatively high educational level and a high SES. This means that our findings are not automatically applicable to other children (and parents), i.e. from other nationalities, with lower educational levels, and/or with lower SES. We suppose that higher educated/SES parents generally may be more aware of typical development and behaviour, and changes or deviations therein. Probably, they may better understand the importance of screening and research, and may also be more inclined to search for help than lower educated/SES parents. However, these assumptions should be tested to make reliable statements. The chance to develop deviant behaviour is influenced by predisposing factors, like genetic constitution, problems during pregnancy or birth, developmental delays or psychiatric disorders in siblings and parents. Regrettably, information about such factors was not available, hence it was not possible to investigate the role of these factors on the children’s behaviour. Unfortunately, only one parent report per child was available and information from additional sources was lacking, because the children were not clinically assessed. At this young age parents are important persons to signal problems in their children, both in the general population and in clinical samples [6,12,16,19]. However, parental incidental observations and reports are not as accurate and effective as professional standardized procedures and measurements to indentify children’s symptoms. Above that, parents and professionals seem to detect different aspects of abnormal behaviour [39,40]. Thus, it is desirable to incorporate observations, measurements, and reports of both informants to increase the sensitivity and specificity in future research.

The behavioural classes identified in this study were based on statistical analyses of parent reports, and not on theoretical knowledge and/or clinical experience. We assumed that the children in classes 1, 2, and 4 had relatively normal behaviour, whereas the children in the other classes showed deviant behaviour in some aspects. However, there appears to be a grey area between normal and deviant behaviour. Based on class prevalence at both time points, one could have referred to class 2 as the normal class instead of class 1. The lively and mild negative behaviour of class 2 could have been considered as developmentally typical, whereas the quiet and obedient behaviour of class 1 could have been interpreted as slightly different. Further research may shed more light on the exact type and cause of these variations. Strict factorial invariance, and thus configural (measurement) invariance, is needed for comparisons of factor scores between classes [10]. Because we did not specifically test for it, one should be cautious when comparing factor scores across classes at the same time point. However, invariance seems to be of minor importance and influence when comparing classes over a certain time span as is done in the transition model.

We used FMM to determine factors and classes at two time points and we established the transition model afterwards in a separate step. If one was only interested in the division of children into homogeneous groups and the transitions over time, latent transition analysis (LTA), in which the computation of classes and transitions is integrated into one model, may have been an adequate alternative. However, if one preferred a complete picture of both factors and classes, then FMM would have been more appropriate. The factors point in the direction of certain types of behaviour and/or child psychiatric disorders of various groups of children. Depicting the division of and the mutual relations between classes at several time points gives insight in longitudinal transitions. Careful predictions about chances of improvement and risks of problems in the transition from infancy to toddlerhood can only be made for groups of children and not for individual cases. At the group level, there was no significant transition from completely normal behaviour to deviant behaviour and vice versa. Nevertheless, such changes may have occurred in some children. This is of minor relevance in epidemiological research, but of major importance in clinical practice. The normalization of behaviour in children with severe problems may have been overestimated, because there was selective attrition of the two most impaired classes, a commonly observed phenomenon in this type of research [41]. However, this only further underlines the persistence of behavioural difficulties from infancy to a later age.

## Conclusion

The results suggest that certain problems may persist from infancy to toddlerhood, while others may change into other problem behaviour or may disappear. Profound communication and interaction problems as well as negative and demanding behaviour in infancy often result in mild to severe problems in toddlerhood, with the former being least transient and the most specific predictor. More research is necessary to determine the relationship between behavioural problems in the classes identified and in clinical diagnoses.

## Abbreviations

ADHD: Attention-deficit/hyperactivity disorder; ANOVA: Analysis of variance; ASD: Autism spectrum disorder; DC 0-3R: Diagnostic Classification of mental health and developmental disorders of infancy and early childhood, revised edition; DSM-IV-TR: Diagnostic and Statistical Manual of mental disorders, fourth edition, text revision; EFA: Exploratory factor analysis; ERV: Estimated residual variance; ESAT: Early Screening of Autistic Traits Questionnaire; FMM: Factor mixture modelling; ICD-10: International Statistical Classification of Diseases and related health problems, tenth revision; LCA: Latent class analysis; LMR adj. LRT: Lo-Mendell-Rubin adjusted likelihood ratio test; ODD: Oppositional defiant disorder; RMSR: Root mean square residual; SBQ: Social Behaviour Questionnaire; SES: Socioeconomic status; (SSA) BIC: (Sample-size adjusted) Bayesian information criterion; USQ: Utrecht Screening Questionnaire; VLMR LRT: Vuong-Lo-Mendell-Rubin likelihood ratio test.

## Competing interests

In the past five years, Jan K. Buitelaar has been a consultant, advisory board member, and/or speaker for Janssen Cilag BV, Eli Lilly, Organon/Shering Plough, UCB, Shire, Medice, and Servier. He is not an employee or a stock shareholder of any of these companies. He has no other financial or material support, including expert testimony, patents, and royalties. The other authors declare that they have no competing interests.

## Authors’ contributions

EM was responsible for collecting and analysing the data as well as writing the manuscript. ML performed statistical analyses, interpreted data, and commented on drafts. SS designed and coordinated the study and supervised the data collection. NL and JB reviewed and edited subsequent drafts. All authors read and approved the final manuscript.
